# Medical students create multiple-choice questions for learning in pathology education: a pilot study

**DOI:** 10.1186/s12909-018-1312-1

**Published:** 2018-08-22

**Authors:** Rebecca Grainger, Wei Dai, Emma Osborne, Diane Kenwright

**Affiliations:** 1 Department of Pathology and Molecular Medicine, University of Otago Wellington, Wellington, New Zealand; 2 Higher Education Development Centre, University of Otago Wellington, Wellington, New Zealand

**Keywords:** Student-generated MCQ, Multiple-choice questions, Assessment for learning, PeerWise, Bloom’s taxonomy, Peer-instruction, Medical students

## Abstract

**Background:**

Medical students facing high-stakes exams want study resources that have a direct relationship with their assessments. At the same time, they need to develop the skills to think analytically about complex clinical problems. Multiple-choice questions (MCQs) are widely used in medical education and can promote surface learning strategies, but creating MCQs requires both in-depth content knowledge and sophisticated analytical thinking. Therefore, we piloted an MCQ-writing task in which students developed MCQs for their peers to answer.

**Methods:**

Students in a fourth-year anatomic pathology course (*N* = 106) were required to write MCQs using the PeerWise platform. Students created two MCQs for each of four topic areas and the MCQs were answered, rated and commented on by their classmates. Questions were rated for cognitive complexity and a paper-based survey was administered to investigate whether this activity was acceptable, feasible, and whether it promoted desirable learning behaviours in students.

**Results:**

Students were able to create cognitively challenging MCQs: 313/421 (74%) of the MCQs which we rated required the respondent to apply or analyse pathology knowledge. However, students who responded to the end-of-course questionnaire (*N* = 62) saw the task as having little educational value. Students found PeerWise easy to use, and indicated that they read widely to prepare questions and monitored the quality of their questions. They did not, however, engage in extensive peer feedback via PeerWise.

**Conclusions:**

Our study showed that the MCQ writing task was feasible and engaged students in self-evaluation and synthesising information from a range of sources, but it was not well accepted and did not strongly engage students in peer-learning. Although students were able to create complex MCQs, they found some aspects of the writing process burdensome and tended not to trust the quality of each other’s MCQs. Because of the evidence this task did promote deep learning, it is worth continuing this mode of teaching if the task can be made more acceptable to students.

## Background

Faced with high-stakes examinations, medical students study strategically. They look for ways of consolidating their knowledge of the core curriculum and prioritise study materials and strategies that relate directly to their upcoming exams [1]. Because medical education makes extensive use of MCQ exams, many students preparing for multiple choice examinations therefore tend to favour multiple-choice question (MCQ) question-banks. These resources engage students in practice-test-taking to consolidate knowledge but the majority of MCQs test lower-order thinking skills (recall and comprehension) rather than higher-order skills such as application and analysis [2, 3].

In order to write MCQs, students need to use higher-order thinking skills [4]. This challenging task requires deep understanding of the course content and thoughtful answering strategies [5]. In a question-generating process as a learning exercise students are required to process, organize, integrate and reconstruct knowledge, which improves meta-cognitive development and encourages higher-order thinking [6–10]. Moreover, by evaluating and providing critical feedback on questions generated by peers, students may engage in collaborative learning, which encourages self-reflection, communication and problem-solving skills [9, 11–14]. Medical students have found these kinds of student-generated question-banks to be valuable learning resources [15]. Student-generated questions can also highlight when students have a flawed understanding of the course material more effectively than students’ answers to MCQs, and thus provide a formative opportunity to address misconceptions [16]. Requiring students to write MCQs may therefore develop these desirable problem-solving and collaborative skills while engaging students in a task that has immediate and clear relevance to their high-stakes MCQ assessments.

PeerWise is a free web-based platform for students to create, answer, and review MCQs [17]. As an entirely student-driven system with minimal instructor input, PeerWise may engage students through the “writing to learn” process and supports student ownership of their learning environment [18–20]. PeerWise incorporates gamification with leader boards for writing, answering and commenting and “badges” for achieving participation milestones. PeerWise has been widely used in educational institutions, with reported enhanced student engagement and correlations described between higher PeerWise activity and improved academic performance [21–23]. While using student-generated MCQs for learning can enhance educational outcomes, the design of student-written MCQ tasks appears to affect whether they lead to surface learning or foster desirable learning strategies. Some studies which have included student-written MCQs in summative assessments found that lead to rote memorisation [24] or failed to improve students’ learning strategies [25]. Therefore, it is important to monitor whether introducing MCQ-writing does indeed foster deep learning strategies.

There has been a call for further research into the quality of student-written MCQs [21]. Previous studies have found that the majority of items in biology and biochemistry student-generated question banks draw on lower-order thinking skills [9, 20]. Other studies found that medical students wrote scenario-based questions at a lower rate than faculty members [26] and needed multiple attempts to create higher-order questions [24]. Therefore, one aspect of MCQ-writing that needs to be investigated is whether it is feasible to design an MCQ-writing task that can draw on higher order thinking in both writing and answering MCQs.

## Methods

Because previous research into PeerWise has not explored complex MCQs extensively, we used a pilot study approach [27, 28] to assess whether it was feasible, acceptable to students and engaged students in desirable learning behaviours.

### MCQ-writing task

Students were asked to write MCQs in four modules of a fourth-year anatomic pathology course (cardiovascular, central nervous system, respiratory and gastrointestinal). For each module, each student was required to create at least two MCQs and correctly respond to at least twenty peer-generated MCQs. Peer feedback evaluating MCQs, by rating and commenting on the question or the explanation, was strongly encouraged but not required. .

Each MCQ was required to comprise a stem, one correct answer and three or four plausible distractors. Provision of detailed explanations justifying the correct option and explaining thinking behind the distractors were required. MCQs were tagged to each topic area within PeerWise. Students rated MCQ quality on a six-point scale (with descriptors of 0 very poor, 1 poor, 2 fair, 3 good, 4 very good, 5 excellent). The “Answer Score” within PeerWise was used to track correct MCQ answering. The Answer Score rewards students with 10 points whenever a correct answer is chosen, while a small number of points are deducted for an incorrect answer (depending on the number of options associated with the question) [29]. Thus students needed to complete at least 80 questions (20 per module) to obtain the required Answer Score of 800. Students received 20% of their final grade for the course for completing the PeerWise activity. Half of this mark was for achieving an Answer Score of 800. The other half of the mark was designed to reward generation of high quality MCQs and depended on an external quality rating of one of each students’ MCQs in each module. The 80% balance of the student’s final grade came from a two-hour online examination consisting of 100 single-correct answer MCQ, administered at the end of the academic year.

Students attended a 30-min instructional scaffolding session before the implementation of PeerWise, comprising the pedagogical rationale of the student-generated MCQ approach and technical support to PeerWise system. A main focus of the scaffolding session was to provide guidance regarding how to write high-quality MCQs involving higher-order thinking. Examples of complex and recall-based MCQs, as well as Bloom’s Taxonomy of different cognitive levels were introduced to students [4]. Since we aimed to foster peer-learning and collaboration rather than competition, the gamification features of PeerWise were not discussed during scaffolding or noted in instructional material. A one-hour session of class time was timetabled for MCQ authoring and/or answering for each module, occurring within one to two weeks of relevant face-to-face teaching (lectures and small group tutorials). The activity was open for one semester with a closing date.

### Participants

One hundred and six fourth-year medical students of University of Otago Wellington were enrolled in PeerWise. The Otago Medical School MBChB programme is six years in duration: a foundation year in health sciences, years two and three cover biomedical sciences and introduce clinical practice and years four to six are clinically-based learning. Participation in the MCQ-writing task was compulsory and contributed to students’ summative grade but participation in the research project was voluntary. Students voluntarily participated in the research by completing the end-of-course survey and consenting to allowing their questions to be used as examples. The research was approved by Departmental approval process and subsequently ratified by the Human Ethics Committee of the University of Otago (Category B) and written consent was obtained from students.

### Evaluation

We investigated whether the MCQ-writing task was acceptable to students, whether they could feasibly complete it and whether it engaged students in desirable learning behaviours. A paper-based post-course survey comprising validated assessment tools and free-text questions was used to evaluate student engagement. We also rated students’ questions for cognitive complexity and their comments for depth of participation in a learning community.

### Acceptability

To assess acceptability of the task, we used subsections from two existing surveys. Perceived educational value of the MCQ-writing task was evaluated using the Survey of Student-Generated MCQs (Cronbach’s α = 0.971) [30, 31]. Acceptance of PeerWise was measured using the Technology Acceptance Model (Cronbach’s α = 0.896) [32]. All variables were measured by a seven-point Likert scale (1 = strongly disagree, 7 = strongly agree).

### Feasibility

To assess feasibility of MCQ-writing, we rated MCQs for cognitive complexity and asked students how they went about completing the MCQ task. We rated question quality using a three-level rubric based on Bloom’s taxonomy (summarized in Table 1, see [33] for development of the rating system). We also asked students to indicate how long it took them to complete the task, and asked free-text questions (see Table 2).

**Table 1:** Rubric for assessing MCQ complexity

Level	Corresponds to Bloom’s Taxonomy	Description
Level 1	Knowledge & comprehension	Knowing and interpreting facts about a disease, classification, signs & symptoms, procedures, tests.
Level 2	Application	Applying information about a patient (signs & symptoms, demographics, behaviours) to solve a problem (diagnose, treat, test)
Level 3	Synthesis & evaluation	Using several different pieces of information about a patient to understand the whole picture, combining information to infer which is most probable.

**Table 2:** Free-text questions on feasibility of MCQ writing task

Based on your experience of writing MCQs:	
1. What difficulties did you encounter in writing MCQs? How did you overcome these difficulties?	
2. What would you change about the way this activity was designed?	
3. Did you refer to the MCQ writing guidance that was introduced in the first class?	
4. How did the guidance help you generate your MCQs? Was it useful to prepare you for MCQ writing?	
Based on giving feedback to others and reflecting on your own questions:	
5. What made for a clear MCQ?	
6. What made for a good distractor?	
7. What kinds of questions made you draw on your knowledge of different parts of the medical curriculum?	

### Desirable learning behaviours

We defined desirable learning behaviours as: synthesising knowledge from multiple sources to complete the MCQs; evaluating and improving the quality of students’ own MCQs; and participating in a community of practice with their peers. To investigate knowledge synthesis and self-evaluation, we asked free-text questions (see Table 3).

**Table 3:** Free text questions to evaluate desirable learning behaviours

Based on your experience of writing MCQs:	
1. What sources (e.g. texts or other resources) did you use to develop your MCQs?	
2. Do you think your approach to writing MCQs improve over the semester?	
a. If so, how did it change?	
b. If not, why not?	
3. How did you check that you had included higher order levels of Bloom’s taxonomy?	
4. How did you check that your questions were clearly written?	

We assessed students’ participation in a community of learning using part of the Constructivist Online Learning Environment Survey (Cronbach’s α = 0.908) [34], which was measured on a seven-point Likert scale, and by evaluating the comments students made on each other’s questions. These comments were evaluated using a three-level rubric [5]. Level one comments were phrases (such as “Good question”, “Great Explanation”), Level two comments contained phrases of a scientific nature but no discussion, and Level 3 comments suggested improvements, new ideas or led to further discussion.

### Data analysis

Summary statistics for quantitative data analysis were calculated using IBM SPSS (version 22). Extended responses to open questions were analysed by two of the authors (EO, WD) using thematic content analysis [35, 36]. Where students responded briefly to the open questions (such as responding with a yes/no without elaboration) these responses were analysed numerically.

## Results

Ninety-two students gave consent to participate in the research component and sixty-two students responded to the survey (67% response rate). The mean age of respondents was 22.63 ± 2.1 years old. There were 58% females (36/62), 39% males (24/62).

### Acceptability

**Fig. 1:**
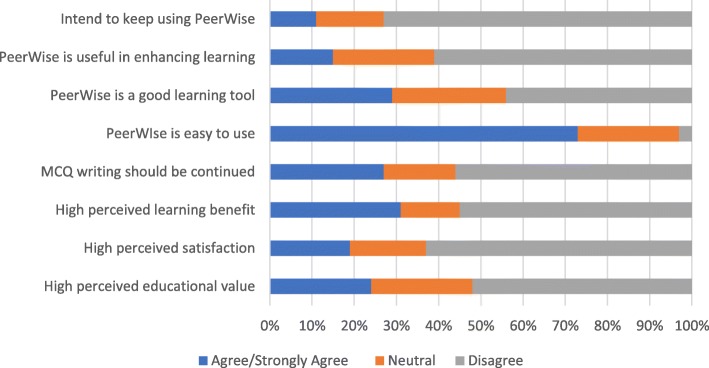
Student perceptions of PeerWise and MCQ writing

Students’ responses to the survey showed a negative attitude towards writing MCQs. Only 24% (15/62) of students agreed (combined Likert scale 5–7) they perceived high educational value of the MCQ writing process, and 22% (14/62) of students agreed that MCQ writing improved their learning experience. Eighty-one percent (50/62) of students were not satisfied with the MCQ writing process, and only 27% (17/62) of students agreed that MCQ writing should be continued in the future. Only 31% (19/62) of students agreed writing MCQs was beneficial to their learning (see Fig. 1).

Although 73% (45/62) of students agreed that PeerWise is easy to use, 61% (38/62) of students did not perceive PeerWise as useful in enhancing their learning. Only 29% (18/62) of students agreed that PeerWise is a good learning tool, and 11% (7/62) of students agreed that they intend to keep using PeerWise.

### Feasibility

Students were largely capable of writing complex, scenario-based MCQs. Expert rating was undertaken on 421 MCQs: 74% (313/421) of the questions were classified as cognitively challenging (Level 2 or 3) involving knowledge application and evaluation, such as arriving at a diagnosis based on a patient scenario, making treatment recommendations and anticipating expected findings of investigations. Only 26% (108/421) of MCQs were classified as level one questions. Table 4 shows the distribution of MCQ quality in each module.

**Table 4:** Cognitive complexity of student-generated MCQs per module

Module	Level 1	Level 2	Level 3
Cardiovascular	34 (32%)	35 (33%)	37 (35%)
Respiratory	19 (18%)	50 (48%)	36 (34%)
Central nervous system	18 (17%)	46 (44%)	41 (39%)
Gastrointestinal	37 (35%)	36 (34%)	32 (31%)

Students were asked to estimate how long they spent writing each MCQ; 8% (5/62) of students competed the task in under 30 min, 51% (32/62) in 30 min to 1 h, 26% (16/62) in 1 to 2 h, and 15% (9/62) in more than two hours.

Open-ended text feedback from the survey indicated that students did not refer to the guidance they were given throughout the semester, preferring to instead check their questions with peers, read over them themselves or incorporate elements of situations that they had experienced themselves in order to create case-based questions. Students generally did not find the guidance they were given on preparing MCQs using Bloom’s taxonomy to be helpful.

### Desirable learning behaviours

The MCQ-writing task engaged students in reading widely and synthesising information from multiple sources. Most respondents named two or more different resources they had used to write their MCQs. Fifteen percent (9/59) of students identified a single source used to complete the task, 51% (30/59), identified two sources, 22% (13/59) identified three sources and 12% (7/59) more than three sources. Most students drew on the lecture material and at least one other source of knowledge. The most popular named sources that students used in addition to their lecture material were the set textbook, their e-learning tutorials for pathology and an additional recommended text. Students also used a range of online sources aimed at both clinicians and consumers.

Free text survey responses indicated students checked the clarity of their questions by reading over their own questions, asking peers to read their questions before posting, and looking at feedback and ratings after posting. Students generally reported that they did not refer to Bloom’s taxonomy to monitor the cognitive complexity of their questions but used other strategies such as choosing a style of question (e.g. multi-step or case-based) that lent itself to complex thinking, getting feedback from peers reading over the questions themselves.

Fifty-two percent of the respondents (27/52) believed that their approach to writing MCQs had improved over the semester and 48% (25/52) said it had not. Respondents identified in the free-text question that over the course of the semester the process of writing MCQs became easier or quicker, and the questions they wrote were clearer, more sophisticated and better aligned with the curriculum. A few students reported that over the semester they wrote less sophisticated questions.

Most students participated to at least some degree in a peer community by commenting but students tended not to value these comments, and only a few students participated extensively or deeply in commenting on each other’s questions. Thirty-six percent of MCQs (320/885) received at least one comment. Of the total 843 comments, 34% (287/843) were classified as level three comments (leading to discussion of, or improvements to the MCQ), while 59% (497/843) of comments were classified as level one comments (e.g. “*good question”* or “*nice explanation”*). The majority of the comments were made by just 23 students, all of whom submitted more than ten comments each. Only 11 students did not participate at all in commenting. Seventy-two percent (76/106) of authors responded to comments on their questions.

Students’ responses showed they did not value this peer learning. Only 24% (15/62) of students agreed that other students valued their contribution and only 37% (23/62) of students agreed that collaboration with peers was beneficial. Students also reported low perceived value of the peer learning environment, with 68% (42/62) of students reporting they did not interact with their peers about their MCQs. For students that did report peer interaction via comments, 29% (18/62) of students reported asking others to explain their MCQs or the MCQ answers and 24% (15/62) students reported they were asked for explanations. Only 19% (12/62) of students agreed that commenting on MCQs was beneficial for learning and 31% (19/62) students reported making good use of the comments received on their MCQs or commented on others’ MCQs. Specifically, 31% (19/62) of students reported making good use of comments received, and 40% (25/62) of students reported that other students made good use of their comments (such as correcting mistakes or improving explanations).

## Discussion

This pilot study exploring the implementation of student-written MCQs found that the majority of students successfully wrote questions that tested application and analysis of pathology. Most participants also engaged in the desirable learning behaviours of self-evaluation and synthesising information from a range of sources, but the majority of students did not see the educational value of the activity or participate deeply in a community of learning in PeerWise.

### Relationship to previous literature

The quality of student-generated MCQs suggests students were engaged in active learning and deepened their understanding of learning materials [20]. This is consistent with previous literature measuring question complexity in PeerWise [9, 37]. This can also be inferred from students’ feedback on the learning activities associated with the question-generating process: Students reported spending time on collecting and synthesising information about the particular topic from multiple sources before generating each MCQ, as well as a sense of improvement of MCQ writing over time. Other authors have also reported high student engagement in learning when students were actively involved in the creative process of constructing MCQs compared with passively answering MCQs [17, 20, 38]. In this pilot study the student-generated MCQ approach was introduced to students as an active learning opportunity rather than as an online practice tool. The MCQ-generation process actively engaged students, which not only reinforced concepts learned in class, but also developed deep learning including knowledge analysis, evaluation, and creation.

In contrast to most previous literature [20, 21, 30, 39], the MCQ-generation process was perceived negatively by students. Although the “learning by doing” rationale of the self-generated MCQ approach was introduced to students during the scaffolding session, it is possible that students did not recognise all the indirect learning benefits of writing MCQs such as improving their higher-order thinking skills. Although many students perceived this educational initiative negatively and most students contributed the minimum number of MCQs, many students answered more MCQs than required. By answering MCQ students may quickly identify gaps in their knowledge which they may consider valuable for directing future learning [18, 40]. Students appear to be driven by the expectancy-value theory; they see short-term benefit of answering MCQs but not the long-term benefit of generating them [1, 41].

Although an online peer-based learning opportunity was available within the PeerWise platform, only a small fraction of students actively engaged in a peer learning process. Most students did not feel their contribution was valued by peers, and considered they did not benefit from collaborative learning as educationally useful feedback was provided infrequently. It is possible students perceived instruction and feedback from peers as less convincing or reliable than that from experts. Students have been found to be uncertain about the knowledge and expertise of their peers in other peer-instruction environments and to doubt the reliability and correctness of peer-generated questions and explanations [42–44]. In medicine this maybe a particular problem due to the strong hierarchy and apprenticeship model. Students may not have trusted each other enough to rate questions fairly. With trust and safety being an essential component of a peer learning community, students may be reluctant to expose themselves, even anonymously.

### Implications for teaching

This pilot study focused on acceptability and feasibility of the student-generated MCQ in healthcare professional education, hence evaluation of the impact on learning by correlation of participation or MCQ-quality and performance in examinations or other assessment was not undertaken. This learning intervention is feasible, evinced by students finding PeerWise easy to use and completing the required tasks. However, it was not acceptable to the majority of students, given the negative perceptions of the task. It is possible that the high cognitive load [45] required for task completion seemed excessive to students, contributing to negative perceptions of the task. Furthermore, the MCQ-writing exercise was delivered in the first half of the year, concurrent with anatomic pathology teaching, however the examination occurred at the end of the year. This may have contributed to negative perceptions of the task as immediate relevance to summative assessment may not have been clear and concurrent clinical-based learning may have taken time priority.

In future iterations of student MCQ-writing, instructors should aim to minimise the extraneous cognitive load associated with the task and increase students’ confidence in the quality of their peers’ questions. Using Bloom’s taxonomy [4] as a model for question-writing may introduce extraneous demands on students; the students in this study tended not refer to the guidance on Bloom’s taxonomy during the semester, preferring peer feedback and looking at other questions as models. Therefore, a better approach may be to model and direct students to write scenario-based clinical questions without introducing Bloom’s taxonomy. To increase students’ confidence in the quality of their peers’ questions and to reduce the extraneous cognitive load of thinking of suitable topics for MCQs, topics could be assigned directly to students. This would ensure that the question bank as a whole covered the core pathology curriculum evenly and may therefore increase the perceived value of the question bank as a revision resource. Once a format of student MCQ-writing that is acceptable to students has been established, both evaluation of learning using objective measures like examination results and analysis of MCQ item statistics would be highly worthwhile. Furthermore, instructors could select high-quality student-generated MCQs for inclusion in final summative examination, which may provide additional incentive for student participation and increase acceptability of the activity.

### Limitations

This study has several limitations, one of them being the relatively small sample size (106 participants). Thus, the current findings might not be generalized to the broader healthcare profession education. Additional studies utilising larger sample size and different settings are required. Another limitation of this study is the participation in the evaluative research; only 86% (92/106) of students consented to participate in the research and only two-thirds of these students (62/92) completed the survey. Hence a representative sample might not be guaranteed and no-response bias might exist. Expert rating of student MCQs was only performed on questions that received better peer rating to encourage student participation. This may have introduced a positive skew to the MCQ-quality rating leading to an over-estimation of overall MCQ quality and thus over-estimation of the educational value of the intervention.

## Conclusion

The student-generated MCQ approach implemented in this anatomic pathology course appeared feasible although not acceptable to students. Although students did not enjoy the challenging MCQ-generating process, the quality of the question repository and reported problem-solving strategies may indicate engagement with the course material. An online peer-instruction environment with peer learning through constructive discussions was only partially achieved. Some students expressed concerns about the expertise of their peers, as well as about the accuracy of peer-generated MCQs. Future iterations of this intervention should consider reducing the perceived demands of the task or require scaffolding by instructors to increase students’ confidence in the quality of peer-generated MCQs and to facilitate a more active peer learning environment. Once a feasible and acceptable intervention is established exploration of impact on objective measures of learning and item statistics of the student-generated MCQ would be valuable.

## Abbreviation

MCQ: Multiple-choice question
